# Snakebite-associated audiological sequelae: Rethinking care pathways in African health systems

**DOI:** 10.4102/jphia.v17i1.1919

**Published:** 2026-07-14

**Authors:** Suvishka Barath, Ntandoyenkosi L. Msomi, Andrew J. Ross

**Affiliations:** 1 Department of Family Medicine, College of Health Science, University of KwaZulu-Natal, Durban, South Africa; 2 Department of Public Health Medicine, College of Health Sciences, University of KwaZulu-Natal, Durban, South Africa

**Keywords:** snakebite envenomation, hearing loss, vestibular dysfunction, audiology, neglected tropical diseases, public health

## Abstract

Snakebite envenomation remains one of the most neglected tropical diseases (NTDs), with clinical management primarily focused on survival and acute complications. While mortality and limb-related disability have been well documented, potential auditory and vestibular sequelae remain underexplored, particularly within African health systems. Emerging evidence, primarily from non-African settings, suggests that snakebite survivors may experience hearing loss and vestibular dysfunction; however, these outcomes are not routinely recognised or integrated into rehabilitation pathways. This article provides a conceptual public health perspective informed by existing literature and implementation considerations for hearing healthcare in snakebite management. It examines potential mechanisms of snakebite-associated audiological dysfunction and identifies gaps in surveillance, research and post-acute care within sub-Saharan Africa. We propose the integration of task-shifted and technology-supported audiological screening, referral pathways and rehabilitation services into snakebite care frameworks. Strengthening surveillance and incorporating functional outcomes into national NTD strategies may improve post-snakebite disability prevention and rehabilitation outcomes across African health systems.

## Introduction

Snakebite envenomation represents one of the most neglected global health emergencies, disproportionately affecting impoverished rural populations who have limited access to medical infrastructure and rehabilitation services, particularly those in lower-middle-income countries.^1^ Since the classification of snakebite envenoming as a Neglected Tropical Disease (NTD) in 2017, and the subsequent launch of *Snakebite Envenoming: A Strategy for Prevention and Control* in 2019,^2^ progress has been made in antivenom research and acute management. Nevertheless, the long-term and non-lethal complications of envenoming continue to receive minimal scientific and policy attention. Among these, auditory and vestibular sequelae may represent an unrecognised but clinically significant dimension of post-envenomation morbidity, particularly in regions with high snakebite incidence and limited access to audiological care.

Sub-Saharan Africa (SSA) contributes to more than 30% of global snakebite-related mortality annually, highlighting the magnitude of this public health burden. Snakebites are particularly prevalent in agrarian communities, often resulting in chronic complications, such as blindness, amputation and neuropathic pain.^3,4^ Despite this, auditory and vestibular complications remain conspicuously absent from African epidemiological data. This lack of literature likely reflects under-investigation and limited clinical recognition rather than a true absence of pathology, thereby perpetuating the marginalisation of auditory health within NTD frameworks. While this discussion is situated within the broader sub-Saharan African context, South Africa is referenced as an illustrative case example of how emergency-oriented snakebite services operate within relatively well-resourced health systems in the region.

This article explores emerging evidence linking snakebite envenomation to auditory and vestibular dysfunction and argues for the integration of audiological surveillance and rehabilitation into existing snakebite response frameworks in African health systems.

## Potential mechanisms of ototoxicity and vestibular involvement

Hearing loss and balance dysfunction following snakebite have been sporadically documented, mainly in Asia.^5,6^ The pathophysiological mechanisms underlying these manifestations are likely multifactorial, encompassing direct ototoxic effects, neurovascular injury and systemic inflammatory responses. Venoms from snake families such as *Atractaspididae, Elapidae, Hydrophidae* and *Viperidae* contain complex mixtures of enzymatic and non-enzymatic proteins such as phospholipases A_2_, metalloproteinases, neurotoxins and cytotoxins.^7,8,9^ These bioactive components may contribute to widespread neurotoxicity, disrupt cochlear microcirculation and trigger oxidative stress cascades that damage sensory hair cells, potentially resulting in sensorineural hearing loss.^10,11^

Case reports of krait bites have described sudden bilateral sensorineural hearing loss, confirmed by pure tone audiometry, auditory brainstem responses and otoacoustic emissions.^5,6,12^ These findings raise the possibility that inner ear structures may be affected following envenomation. Similarly, a study from Korea documented vertigo in a viper-bite survivor, raising the possibility that envenoming may precipitate vestibular inflammation through proinflammatory mechanisms rather than direct neurotoxicity.^13^ Taken together, these findings suggest a possible association between snakebite envenomation and audiological or vestibular dysfunction. However, the current evidence base remains limited largely to isolated case reports and small observational studies originating predominantly from Asian contexts. Consequently, the mechanisms, prevalence, timing and causal pathways underlying these manifestations remain poorly understood, particularly within sub-Saharan African populations where epidemiological evidence is currently absent.

In addition, the temporal relationship between envenomation, antivenom administration and the onset of audiological symptoms remains unclear. While venom-related neurotoxicity and inflammatory mechanisms have been hypothesised, treatment-related factors, including potential adverse effects associated with antivenom administration, cannot be excluded based on the currently available evidence.

Accordingly, the present article should be interpreted as a conceptual and public health systems discussion intended to highlight an underexplored area requiring further epidemiological and implementation-focused investigation within African contexts.

## Health system and research gaps in post-snakebite care

Despite growing recognition of snakebite as an NTD, African research has remained largely silent on its potential audiological sequelae. The lack of surveillance and post-acute follow-up protocols means that subtle, but functionally significant impairments such as hearing loss, tinnitus or vertigo, go undocumented and untreated.

National guidelines addressing disabilities associated with NTDs are essential for delivering inclusive, patient-centred care. Such frameworks should ensure that health systems are equipped to identify and manage the long-term physical, psychological and social consequences of disease. In alignment with global targets, the World Health Organization’s 2021–2030 Road Map for Neglected Tropical Diseases calls for countries to develop and implement national guidelines addressing at least 75% of the NTDs endemic within their borders.^1^ However, current national NTD strategies rarely consider auditory or vestibular complications that may accompany certain infections or envenomation, representing a missed opportunity for disability prevention and rehabilitation.

Access to effective care for snakebite envenomation remains unequal across SSA, where persistent antivenom shortages undermine timely and evidence-based management. The limited availability of safe and effective antivenom in many regions often leads individuals to seek care from traditional healers, particularly in rural areas where formal healthcare infrastructure is sparse.^14^ While traditional healing practices remain an important component of community healthcare, delayed presentation to medical facilities may increase the risk of systemic and neurological complications. As a result, subtler long-term outcomes such as hearing loss may remain unrecognised within both clinical practice and public health surveillance systems. Addressing these access challenges is therefore critical not only for reducing mortality but also for identifying and managing the full spectrum of post-envenomation disabilities within affected communities.

## Designing inclusive snakebite care pathways

From a public health service design perspective, the omission of audiological sequelae reflects not only a research gap but also a limitation in the structure of snakebite care systems. South Africa is referenced illustratively to contextualise how emergency-oriented snakebite services may operate within relatively better-resourced health systems in SSA. Within many regional and tertiary healthcare facilities, snakebite care pathways remain primarily centred on emergency stabilisation and antivenom administration, with limited incorporation of structured long-term functional surveillance and rehabilitation.^15^ Designing comprehensive snakebite services, therefore, requires extending care beyond emergency treatment towards functional recovery and disability prevention.

**FIGURE 1:**
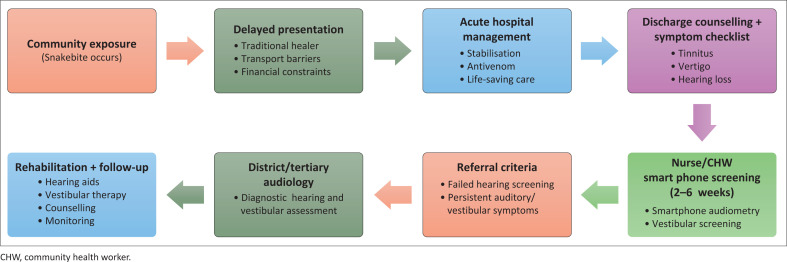
Illustration of the current snakebite care pathway and proposed integration of a tiered audiological screening and rehabilitation pathway.

Snakebite follow-up pathways in resource-constrained African health systems may require a tiered and task-shifted approach rather than reliance on specialist-led assessment alone. Given the limited audiology workforce across many sub-Saharan African settings, universal audiologist-led surveillance is unlikely to be feasible at scale. Initial hearing and vestibular screening could instead be incorporated into routine post-envenomation follow-up using brief symptom questionnaires and validated smartphone-based audiometric screening tools administered by trained nurses or community health workers (CHWs). Validated smartphone-based audiometric screening platforms have demonstrated promising feasibility and accuracy within low-resource hearing healthcare settings and may therefore provide a scalable approach for post-snakebite auditory surveillance in underserved communities.^16^ Individuals presenting with tinnitus, vertigo, communication difficulties or failed screening results could then be referred to district or tertiary audiology services for comprehensive diagnostic assessment and rehabilitation where indicated. Such an approach may improve feasibility, reduce specialist burden and support earlier identification of disability in rural and underserved communities. Figure 1 depicts the current emergency-oriented snakebite care pathway with a proposed integration of a tiered audiological screening and rehabilitation pathway. To operationalise this proposed integration, a tiered post-snakebite audiological care implementation is outlined in Table 1, summarising the key components, implementation enablers and expected system outcomes. This proposed implementation highlights the importance of decentralised screening, task-shifted implementation and integration of audiological care into existing NTD service structures.

**TABLE 1:** Proposed implementation of a tiered post-snakebite audiological care.

Component	Input	Activity	Output	Outcome
Screening	Nurses/CHWs + smartphone tools	Audiological symptom + hearing screening	At-risk individuals identified	Early detection
Referral	Referral pathways and audiology access	Referral for diagnostic assessment	Audiological evaluation completed	Earlier diagnosis and intervention
Rehabilitation	Audiology and rehabilitation services	Hearing aids, vestibular therapy, counselling and follow-up	Functional support provided	Improved communication and quality of life
System strengthening	Policy integration and workforce support	Inclusion within NTD and rehabilitation frameworks	Coordinated care pathways established	More sustainable and inclusive service delivery

CHW, community health worker; NTD, neglected tropical disease.

Failure to screen snakebite survivors’ hearing and vestibular function undermines universal health coverage and access to rehabilitation. Undiagnosed auditory impairment may compound the socioeconomic vulnerability already associated with snakebite exposure in agrarian populations in rural and peri-urban areas. Equitable service designs must additionally account for sociocultural and geographic barriers that influence healthcare access across many sub-Saharan African settings. Given that many snakebite victims initially consult traditional healers or reside in remote agrarian communities, community engagement and health education are essential components of early detection strategies. Raising awareness about potential delayed auditory symptoms may encourage timely presentation and reduce the risk of unrecognised impairment.

Viewed through a public health service design lens, snakebite-associated audiological sequelae represent a systems challenge rather than an isolated clinical phenomenon. Integrating audiological screening, referral and rehabilitation into structured care pathways would not only advance comprehensive snakebite management but also contribute to broader disability inclusion and rehabilitation-strengthening efforts across SSA.

## Implementation priorities

Despite emerging conceptual recognition of snakebite-associated audiological dysfunction, several implementation-focused questions remain unanswered. Future research should establish clinically meaningful changes in hearing thresholds and determine the minimal clinically importance difference for patient-reported commmunication outcomes. The optimal timing of screening also requires investigation, including whether bedside screening during acute admission differs in diagnostic yield compared with delayed follow-up at 2–6 weeks post-envenomation. In addition, health-economic evaluations are needed to determine the feasibility and cost-effectiveness of tiered screening models, including the potential cost per disability-adjusted life year averted through early identification and rehabilitation of hearing loss. Future studies should additionally investigate whether audiological manifestations emerge during acute admission or predominantly during post-discharge recovery. Addressing these questions would support evidence-informed integration of audiological surveillance into snakebite care pathways across resource-constrained African health systems.

## Recommendations for clinical practice, research and policy

- **Clinical implementation:** Pilot post-snakebite auditory screening programmes should be evaluated within tertiary and regional hospitals managing high snakebite volumes in SSA over the next 3–5 years.
- **Research:** Prospective longitudinal studies employing comprehensive audiometric and vestibular assessment are required to determine the incidence, severity and natural history of snakebite-associated audiological dysfunction. Future studies should establish optimal screening intervals, compare early versus delayed assessment following envenomation and identify clinically meaningful changes in hearing thresholds, communication participation and quality-of-life outcomes among survivors.
- **Policy and funding:** Functional outcomes, including hearing, balance, communication, participation and quality-of-life measures, should be incorporated into future antivenom trials and post-envenomation outcome frameworks. National NTD strategies and rehabilitation policies should additionally consider integrating disability surveillance and referral pathways for snakebite survivors as part of broader universal health coverage and rehabilitation-strengthening efforts.

## Conclusion

An exclusively emergency-centred model of snakebite care may be insufficient to address the full spectrum of morbidity associated with envenomation. Reorienting snakebite services towards function-centred care, such as incorporating surveillance, rehabilitation and equitable access to audiological services, would strengthen post-envenomation management and support broader disability prevention and rehabilitation goals within African health systems. Integrating audiological services into snakebite response strategies would also align with the World Health Organization’s goals of reducing disability and improving quality of life among individuals affected by NTD.
